# Trust, place, and polysubstance use shape the feasibility of community-based drug checking in the Southern United States: A mixed-methods implementation study

**DOI:** 10.21203/rs.3.rs-10359475/v1

**Published:** 2026-10-11

**Authors:** Sarah Febres-Cordero, Roesheen McGilvery, Daniel J. Smith, Lydia Vlachou, Abigail (Gale) Pires, Fawaz Shanun, Nadiah Cunningham, Hannah L. Nelson, Mojgan Zare, Lisa M. Thompson, Athena D. F. Sherman, Beatrice King

**Affiliations:** Emory University; Emory University; University at Buffalo, State University of New York; Emory University; Emory University; Emory University; Emory University; Emory University; Georgia Harm Reduction Coalition; University of California, San Francisco; Emory University; Fulton County Board of Health

**Keywords:** COM-B framework, Behavior Change Wheel, drug checking, drug analysis, harm reduction

## Abstract

**Background:**

The unregulated drug supply in the United States (US) is increasingly volatile, with rising contamination by fentanyl and xylazine, contributing to overdose and drug-related morbidity and mortality. Drug checking is a promising harm reduction strategy; yet, little is known about how social trust, place, and patterns of polysubstance use influence the feasibility and implementation of community-based drug checking in the Southern US, where punitive drug policies and institutional mistrust remain prominent.

**Methods:**

We conducted a multiphase, sequential, exploratory, and explanatory mixed-methods, community-engaged implementation study in Fulton County, Georgia. The theoretical framework COM-B (Capability, Opportunity, Motivation–Behavior) was used to examine individual- and community-level determinants of engagement among people who use drugs. Quantitative surveys were administered to people who use drugs (PWUD; N = 164), recruited from syringe services programs, nightlife settings, festivals, and informal housing environments, to assess drug use contexts, drug-checking behaviors, and trust in drug sources, and a subset of participants completed semi-structured qualitative interviews (n = 16). Survey findings were analyzed descriptively and used iteratively to inform qualitative data collection. Qualitative data were analyzed using rapid thematic analysis and integrated across frameworks.

**Results:**

Nearly two-thirds of PWUD in the quantitative sample (62%) reported never testing their drugs despite high awareness of overdose risk. Trust in known drug sources was a central theme reducing the perceived need for testing, while distrust of law enforcement and institutions constrained engagement with formal drug-checking services. Polysubstance use was widespread and intentional, complicating the perceived utility of single-analyte testing tools.

**Conclusions:**

This study demonstrates that while community drug checking is urgently needed in Fulton County, its successful implementation is constrained by stigma, trust in source, and limited drug education. Legal reforms and expanded access to testing tools alone are insufficient to ensure uptake of drug checking.

## Introduction

In April 2023, the White House declared the combination of xylazine and fentanyl in the unregulated drug supply (defined as licit and illicit drugs obtained outside of a prescription) an emerging threat to the United States (US) because of its association with rising overdose deaths and severe morbidity (1). Between 2020 and 2021, the presence of fentanyl-xylazine increased across all US Census regions, with the largest increase in the South (193%) (1). The most recent surveillance data demonstrate the continued contamination of the unregulated drug supply in the Southern State of Georgia in the US. From 2020 to 2022, deaths involving any drug increased by 12%, while xylazine-related overdose deaths increased by 1120%, rising from 15 deaths in 2020 to 116 in 2021 and 183 in 2022 (2). During the same period, the proportion of all reported drug overdose deaths involving xylazine increased from 0.8% (15 of 1,888 deaths) in 2020 to 9% (183 of 2,115 deaths) in 2022, and 100% of suspected xylazine-related overdose deaths in 2022 also involved fentanyl (2).

In August 2021, Fulton County, Georgia, had the state’s highest overdose rate due to an unpredictable, unregulated drug supply (2, 3). The county’s population accounts for one-third of the state’s population, with 1,090,345 residents (4). Fulton County experienced 127 opioid-related deaths in 2020 (3). Most fatal overdoses occurred among Black/African American residents. National projections indicate that although overall overdose death rates showed some stabilization, even a slight decrease between 2022 and 2023, drug overdose rates increased among Black non-Hispanic people, and projections through 2025 suggest that overdose deaths among Black men aged 31–47 are expected to increase by approximately 11% (5).

Xylazine (an animal tranquilizer) complicates overdose response by increasing the risk of prolonged sedation, severe soft-tissue injury, and reduced responsiveness to naloxone, underscoring the urgent need for enhanced surveillance and preventive strategies. Fentanyl, a powerful synthetic opioid, has contaminated both opioid and stimulant unregulated drug supplies in the US. Fentanyl in unregulated stimulants is concerning because stimulant users may be opioid-naïve, putting them at greater risk of opioid overdose (6, 7).In addition to opioid-involved overdoses in Fulton County, Xylazine contamination in the unregulated drug supply (especially in the fentanyl supply) has contributed to increased mortality and harm from unregulated drug use (2). The Fulton County Department of Public Health recently began distributing fentanyl test strips ) to people who use drugs (PWUD). However, it did not identify a plan to communicate trends to PWUD when contaminants were found in the unregulated drug supply.

FTS has been shown to be as reliable as mass spectrometry, the most well-known method for detecting drug samples by measuring molecular mass (8–10). Among people who use opioids, FTS has been shown to change drug use behaviors to reduce harm, including preparing a test shot, changing the route of administration, adjusting the dose, and abstaining (11, 12). Xylazine has been found in the unregulated opioid supply in the US and is of concern for PWUD, as it contributes to hard-to-treat skin ulcers and overdose (13–16). Commonly known as “tranq dope,” xylazine test strips are now available for drug screening. However, few studies in the US have assessed the barriers and facilitators to using xylazine test strips (13–21). This is especially needed in the Southern US, where the rise in the fentanyl-xylazine combination was highest between 2020 and 2021 (193%) (22).

Drug checking services (DCS) provide PWUD with a means to monitor, analyze, and communicate trends from the unregulated drug supply to inform decision-making through drug surveillance efforts (23, 24) In a 2023 systematic review, DCS was facilitated by concerns about fentanyl in the unregulated drug supply and the negative health effects of adulterated drugs (23). These concerns led to modified drug use behaviors and behavioral intentions when engaging in DCS (23). Potential barriers to DCS use include lack of concern, trust in drug sources (dealers), and lack of accessibility to DCS (23). However, of the 90 studies included in the systematic review (1990–2018), only nine were conducted in the US, and none were conducted in the Southeastern US (23).

The accessibility of DCS includes onsite (e.g., harm reduction sites, clubs, festivals, and syringe exchange sites) and fixed-site (e.g., mail-in to lab) services and has the potential to reach PWUD who might not test their drugs. Systematic reviews of DCS published in 2022 and 2023 identified that community-based fixed-site services are valuable for marginalized youth and PWUD (23, 25). In a study conducted in Australia, 851 PWUD were surveyed to assess the acceptability of on-site DCS and fixed-site DCS; 94% of those surveyed said they would use an onsite service and 85% said they would use a fixed site service. Study participants reported a willingness to wait an hour for the results with over half of the participants preferring individual feedback (26). These preliminary results show the promise of engagement with DCS. Additionally, on-site (harm reduction agencies) and fixed-site (lab) services using mass spectrometry and toxicology provide reliable results; however, these services require expertise from the person collecting samples (10). To employ these services, we must engage harm reduction agencies and public health departments that have the expertise to collect samples from PWUD.

In response to the evolving overdose crisis and increasing drug supply contamination, Georgia legislated Senate Bill 6 during the 2025 legislative session, clarifying the legality of drug checking and analysis equipment, including fentanyl and xylazine test strips. This policy change removes barriers that previously limited harm-reduction efforts and supports the broader implementation of drug checking as an overdose prevention strategy (27). The recent passage of Georgia Senate Bill 6 (27) presents a critical opportunity to expand drug analysis as an injury-prevention strategy. The bill specifies that “drug analysis equipment used to determine whether a controlled substance or its packaging has been adulterated shall not be considered a drug-related object,” thereby removing legal barriers to drug checking in the state. Drug checking—including drug testing and drug analysis—is a harm reduction strategy already used by people who use drugs (PWUD) to identify unwanted or unexpected adulterants in the unregulated drug supply, such as unintentional exposure to fentanyl or xylazine (25). Although drug checking has demonstrated the potential to reduce overdose risk and drug-related morbidity and mortality, little is known about the uptake, feasibility, and implementation of drug checking interventions among public health agencies and individuals in Georgia.

## Methods

### Study Rationale and Purpose

This study was driven by community concerns regarding the delayed surveillance of unregulated drug supply in Fulton County, Georgia. Following an overdose cluster in downtown Atlanta, discussions with public health professionals, harm reduction practitioners, and community members highlighted the need for more timely and actionable methods to inform the public about emerging drug-related threats. Existing surveillance systems are perceived as too slow to detect rapid changes in the drug market, limiting their utility for real-time harm reduction and overdose prevention. Fentanyl and Xylazine test strips are becoming more accessible in Georgia as a harm reduction strategy for individuals, particularly those who use stimulants such as cocaine (28). The next step is to deploy DCS in community settings and increase access to high-quality DCS with mass spectrometry and in laboratories (1, 29). The results can be used to create timely and reliable drug alerts for PWUD, thereby reducing harm and mortality from unregulated drug supplies (30). No such DCS is provided for PWUD in Georgia, where the results are reported to communities at risk of drug-related harm and overdose.

In other regions of the US, public health departments and academic institutions have begun to address this gap through community-based drug supply surveillance efforts that actively engage with PWUD. For example, public health agencies in New York City and universities such as Brandeis University in Massachusetts and the University of North Carolina at Chapel Hill have implemented programs that enable individuals to report results from personal drug analyses, thereby contributing to shared surveillance and harm-reduction messaging (25, 31, 32). Brandeis University’s StreetCheck program was a key inspiration for this study.

StreetCheck is a free CDC-funded community drug-checking application that supports three levels of drug analysis (9, 31):

1. Individual-level testing using drug checking test strips,
2. In-house Fourier-transform infrared spectroscopy (FTIR) testing was conducted at harm-reduction agencies or mobile outreach sites, and
3. Laboratory-based testing through academic or public health laboratories.

Individuals who perform drug testing can upload their results to a shared platform accessible to other users, harm reduction organizations, and public health partners, thereby supporting real-time harm reduction messaging and drug supply surveillance. For the purpose of this study, we assessed barriers and facilitators to implementing drug checking using test strips, FTIR, and laboratory-based testing, as well as the potential implementation of a drug-checking application, with StreetCheck serving as the model platform.

### Study Setting, Design, Recruitment, and Participants

The current study is a community-engaged implementation study to examine the feasibility, acceptability, and contextual determinants of implementing a community-based drug-checking application. The design intentionally integrated iterative quantitative and qualitative data collection and centered community expertise through a community liaison–driven recruitment model (33). The theoretical framework COM-B (Capability, Opportunity, Motivation–Behavior) was used to examine individual- and community-level determinants of engagement among people who use drugs (PWUD) Fig. 1; (34–36). The Behavior Change Wheel (BCW) is a theory-informed framework for systematically developing behavior change interventions, centered on the COM-B model, which conceptualizes behavior as arising from interactions among capability, opportunity, and motivation (35, 36). The BCW links these behavioral determinants to nine evidence-based intervention functions. The Theoretical Domains Framework (TDF) complements the BCW by synthesizing constructs from multiple behavior change theories into 14 domains representing key influences on behavior, such as knowledge, skills, social influences, and environmental context (37). Together, the BCW and TDF support the identification of barriers and facilitators to behavior and guide the selection of targeted, theory-driven intervention strategies

The study was conducted in Fulton County, Georgia, and included multiple participant groups: people who access syringe exchange services, those from nightlife settings, festival goers, and those from informal housing settings where people use and sell drugs (trap house). The settings were intentionally diverse to capture variations in trust, access, legal risk, and social context related to drug checking and mobile technology use.

#### Quantitative sample.

PWUD were recruited from syringe services programs (SSP), nightlife settings (including bars in historically Black/African American neighborhoods), festivals (including Black Gay Pride and Southern Fried Queer Pride), and informal housing environments (trap houses, i.e., houses where drug sales are nearly constant throughout the day/night). Inclusion criteria were people who use drugs 18 years or older and had used at least one unregulated drug (outside of a prescription) in the past 12 months. Those who exclusively used cannabis were excluded, as there is insufficient evidence that the cannabis supply has been adulterated with fentanyl and xylazine (38).

Data collection began June 17, 2024, and ended November 11, 2024 (five months over 16 data collection days). 180 PWUD were screened for inclusion; 164 were enrolled (acceptance rate: 91%) (39). Of those who did not meet inclusion/exclusion criteria or chose not to enroll, the most common reasons were that they used only cannabis (n = 12), that they did not want to participate in research (n = 2), and that two participants did not provide a reason.

Recruitment relied heavily on community liaisons, individuals already embedded, trusted, and well-known within their communities (33, 40). Community liaisons do not require formal credentials; their expertise is derived from lived experience, long-standing community presence, and demonstrated commitment to community safety. In several settings, the principal investigator functioned as a *de facto* community liaison, reflecting a broader reality in community-engaged research, wherein investigators embedded in the communities they study assume relational, trust-building, and logistical roles beyond traditional academic responsibilities. Recruitment settings with a community liaison led to 142 of the 164 participants recruited. Setting-specific recruitment approaches included the following:

- Syringe Services Program (SSP) - The PI and study team volunteered weekly for one month prior to recruitment, building rapport with staff and participants, and assessing site readiness. Recruitment occurred approximately four weeks later and yielded approximately one-quarter of the sample (n = 73). It should be noted, n = 37 of these participants identified as people who exchange syringes, the rest utilized SSP for wrap around services.
- Nightlife Settings - Recruitment was supported by a trusted bar liaison with whom the study team had an established relationship through years of naloxone training. Advanced outreach and the use of familiar spaces facilitated successful recruitment across multiple evenings (n = 60).
- Festival Settings - Recruitment relied on flyers and on-site outreach without a community liaison, resulting in low participation and underscoring the limitations of outreach without trust-based relationships (n = 15).
- Informal Housing Recruitment - One interview participant became a situational community liaison, facilitating recruitment in an informal housing setting (trap house) (n = 10).
- Word of Mouth/Snowball – (n = 6).

#### Qualitative Sample.

All quantitative participants were asked whether they were interested in participating in an individual qualitative interview. Inclusion criteria for the qualitative interviews consisted of participation in the survey. However, we purposefully sought to include participants who did and did not report using drug checking supplies, came from diverse backgrounds, and had varying self-reports of trust in the source.

### Survey Instrument, Qualitative Interview Guide, and Data Collection

In collaboration with people with lived and living experience of drug use (PWLLE), harm reduction practitioners, and academic and public health partners, we co-created a structured, interviewer-administered survey. A description of survey development has been reported elsewhere (39). However, briefly, the survey explicitly adhered to the principle of “Nothing About Us Without Us.” (41). Surveys were administered by the principal investigator (PI) and the study team.

Demographic and structural variables assessed via the survey were drawn from standardized common data elements (CDEs) from the Emory University and National Institutes of Health repositories. The survey included Likert-scale items, binary response options, and multi-select questions and concluded with an option for participants to provide contact information if interested in a follow-up qualitative interview. Content covered in the surveys included:

- Drug use behavior, routes of administration, settings of use, and sources of drugs
- Drug checking practices, methods used, barriers, and motivations
- Overdose experiences and harm reduction behaviors, including naloxone access and use
- Risk perception and concern related to fentanyl, xylazine, and other adulterants
- Awareness of harm reduction services and the legal status of drug checking tools
- Technology use and comfort, including mobile app usage; and
- Willingness to engage in drug checking and data sharing, including hypothetical scenarios involving test results and delays in use

Prior to the qualitative interviews, participants received a standardized explanation of drug checking and a brief demonstration video of a drug-checking application (Streetcheck). Semi-structured interviews were conducted using a guide informed by the COM-B model. Including concerns about trust, surveillance, gendered and racialized experiences, and legal risk. Interview guides assessed the following areas.

- Capability to test drugs, including skills, knowledge, and identity-based barriers
- Opportunity to access testing technologies and supportive environments
- Motivation to test drugs, share results, and use drug-checking interventions, including potential application-based interventions
- Trust in services and service providers

#### Ethical considerations.

The study was conducted with Institutional Review Board (IRB) supervision from Emory University (STUDY00006583). All the participants provided written informed consent. Given the sensitive nature of illicit drug use, confidentiality, voluntariness, and participant safety were emphasized across all phases. A certificate of confidentiality was provided by the Centers for Disease Control and Prevention.

### Analyses

#### Quantitative analyses.

The community-driven survey assessed participant demographics, drug use settings, drug sourcing, trust in drug dealers or other sources, drug-checking behaviors, and polysubstance use. Survey development procedures have been described elsewhere (39). Trust was assessed using two 10-point items measuring overall trust in drug sources and trust that the substance sold was the intended substance. Responses were categorized a priori into low (1–3), moderate (4–7), and high (8–10) trust to facilitate interpretability of ordinal Likert-type data, consistent with common analytic practices for survey research (42, 43). Quantitative survey data were summarized using descriptive statistics. All statistical analyses were conducted in R Version 4.5.1 (Appendix A.1).

#### Qualitative analyses.

Qualitative data were analyzed using a rapid thematic analysis and analytic approach (44–46). Two researchers independently triangulated data from the short answer items, individual interview transcripts, and interviewer memoing to identify convergent and divergent themes related to barriers and facilitators to drug checking. Structured summaries were developed to align with COM-B and subsequently aligned with relevant domains of the Theoretical Domains Framework (TDF). This mapping process followed established guidance for integrating COM-B and TDF to support behavioral diagnosis and intervention development. To support analytic rigor and transparency (47), data points were mapped to multiple domains when appropriate, and discrepancies were resolved through discussions among the study team. A theme was considered analytically meaningful when it was represented by at least two distinct participant quotations and observed across interviews rather than isolated to a single case. Mapping and thematic interpretation were refined through iterative individual and group discussions among the principal investigator and qualitative analysts.

### Mixed Methods and Theory-Driven Data Analysis, Integration, and Visualization

Integration occurred at multiple analytical points. First, the quantitative survey findings were used to identify the prevalence and distribution of key constructs, including drug use contexts, drug checking behaviors, and trust in drug dealers or sources. Second, qualitative findings were used to explain and contextualize these patterns by identifying the underlying mechanisms, meanings, and decision-making processes. Third, integration was formalized through the application of complementary theoretical frameworks COM-B and TDF were used to integrate individual-level quantitative and qualitative findings. Using that matrix, the research team developed conceptual “mind maps” to visualize relationships between domains and identify cross-cutting themes (Supplement 1: COM-B/TDF Framework Matrix).

The final conceptual flow diagram (Fig. 1) was adapted from Jatau et al. (2019), repositioning “opportunity” at the center to reflect the study’s focus on feasibility and implementation. Bidirectional arrows illustrate the dynamic relationships between behavioral sources, target behavior, and anticipated outcomes (48). COM-B Analysis findings display the integrated COM-B/TDF behavioral determinants derived from interviews and open-ended survey responses, providing a synthesis of how individual behavior change mechanisms and organizational context jointly shape the feasibility and uptake of drug checking. This analysis serves as a central joint display linking qualitative and quantitative findings across the analytic phases

## Results

### Participant Characteristics

The survey sample consisted of 164 people who used drugs in Georgia, and over three-quarters were Black/African American, over half were men, and among them, about one-third were unemployed. A little over half had completed high school or obtained a GED, and about one-fifth of the sample reported using a syringe services program (SSP) (Table 1). The full set of participant characteristics was published by Shanun et al. (2025). Of the 164 participants, 16 were purposefully chosen for individual interviews.

### Drug Use Settings.

Drug use most frequently occurred at home (72.2%), followed by parties (46.2%), bars or clubs (44.9%), streets (41.8%), and cars (38.6%) (Table 2). The drugs were most frequently obtained from local dealers (75.6%) and friends (54.4%). Polysubstance use was common, with three-quarters of the sample reporting using 2 or more substances (excluding cannabis).

## Findings

Figure 1 presents the integration of the COM-B and TDF frameworks to synthesize individual-, social-, and structural-level factors influencing engagement in chemical drug checking and the sharing of results within communities of PWUD. The findings illustrate that drug checking behavior emerges from the dynamic interaction of capability, opportunity, and motivation rather than from any single determinant.

### Capability.

The psychological capability to engage in drug checking varied substantially across participants. Knowledge of drug checking ranged from anecdotal and experiential methods (e.g., visual inspection, cooking substances, water solubility) to evidence-based practices, such as fentanyl test strips (FTS) and reagent testing. Informational barriers were also evident, including lack of knowledge about how to test and limited access to testing supplies (“don’t know how to test,” “not sure how it works,”), as well as perceptions that drug checking required excessive effort or inconvenience (“too much work,” “extra step,” “don’t want to deal with it”). Finally, some participants described using alternative risk-reduction strategies in place of formal drug checking, such as initiating use with a small amount or using more slowly (“I smoke first,” “I start small,” “I go slow”). Collectively, these findings suggest that decisions not to engage in drug checking are shaped by intertwined perceptions of trust, habit, and familiarity, and the prioritization of other harm reduction strategies.

Participants often relied on information passed through peer networks, with understanding shaped more by lived experiences than by formal education. Skills related to drug checking were more commonly observed among individuals already engaged in harm reduction services, suggesting that exposure to harm reduction settings facilitated capability development.

“When I met you guys at (harm reduction outreach), I didn’t know such a thing (test strips) even existed…there needs to be more education on it. I think there needs to be more resources in the community to provide us with the knowledge of it”.

Additionally, one participant who engages with harm reduction services uses fentanyl test strips to ensure they are receiving the drug they are seeking (fentanyl) “just to make sure I was getting what I was buying, you know, what I was paying for.” This desire for drug checking is not only to test for unwanted drugs, but also for purity and other contaminants.

“[FTS] helps because it’s like life or death…but then I think more people are curious as to like how much of this is real coke, and what is the average on that. Like if I get any bag in Atlanta right now no matter what side of Fulton County I get it on like what are we really talking…how many times is it getting stepped on and with what?”.

Memory, attention, and decision-making processes also influence engagement. Participants described intending to test drugs but forgetting moments of urgency or choosing not to delay use, particularly when experiencing withdrawal or craving. Behavioral regulation was also constrained by the effects of substance use itself. The inability to wait to use drugs after acquisition was a frequently cited barrier to testing, even among those who understood its potential benefits. As one participant noted, “But whose gonna…I don’t know…when you have your drugs you wanna do them right then and there”.

Engagement in drug checking is shaped by uneven psychological capability that reflects not just knowledge gaps, but the interaction of learning environments, habits, and the realities of substance use. Participants’ understanding of drug checking often developed through peer networks and lived experience rather than formal education, leading to a mix of informal, experience-based practices and partial awareness of evidence-based tools like fentanyl test strips.

Capability was higher among those already connected to harm reduction services, suggesting that exposure to these settings builds both knowledge and practical skills. However, even when participants understood drug checking, engagement was frequently undermined by competing priorities and constraints, such as urgency, withdrawal, or ingrained routines, that made testing feel like an impractical “extra step.” In this context, individuals often substituted other strategies (e.g., using small amounts first) that better aligned with immediate needs and habits. Overall, capability is not simply about knowing how to test drugs, but about whether that knowledge can be activated in real-world conditions shaped by access, urgency, and patterns of use.

#### Opportunity.

The opportunity to engage in drug checking is shaped by environmental, social, and structural contexts. Structural access barriers included test strips being “hard to find” and “not available where I am”. Participants emphasized the importance of accessible, trusted testing locations, including harm reduction agencies, pharmacies, community spaces, and mobile or delivery-based models. Another trusted location that was identified was in houses where drugs are sold, purchased, and used, the trap house. “I can get test strips.… I get them from like a trap house or something because they have been having them in there.” The ability to have test strips delivered was described by one participant as a facilitator to delaying use until test strips arrive.

If I have the drugs, I’m gonna do them…if it’s like, ‘let’s wait or let’s drive somewhere’, like people don’t even wanna drive to get alcohol sometimes. But now, it’s like can Go Puff bring me some test strips with my alcohol order, then it’s on and popping. If somebody will bring me the strips, I’ll wait because I have alcohol on the way

While public locations increased accessibility, they also raised concerns about visibility, surveillance, and law enforcement, highlighting the tension between convenience and perceived safety. Participants had concerns that fixed drug checking sites would be surveilled by police from drug purchase to drug checking.

a lot of the time they may misinterpret things, or they are just up to get a number. They are to reach a certain quota, so they are just out to get the numbers and do not really care about the crimes or helping the community, because they’ll probably watch that this person is buying drugs, so they are going to want to watch that person.

Social opportunities play a critical role in this process. Stigma surrounding drug use, and for some participants, intersecting stigmatized identities (e.g., LGBTQIA+), limited their willingness to access services or share results publicly. Privacy and anonymity have repeatedly been emphasized as essential conditions for engagement. One participant noted that being gay in and of itself made accessing drug checking dangerous for them. “It would probably make it harder (to bring drugs to a testing site) because my sexuality, a lot of people don’t get with gay people. A lot of people look at us differently.”

Broader sociopolitical factors, including distrust in institutions and fear of legal consequences, further constrained opportunities, particularly in contexts in which drug checking was perceived as operating on the fringes of legality.

Participant states they would trust a drug checking app and “be inclined to use” it, however, they state

“the only barrier, the reason I would not consistently use it is because, I could even put up with the inconvenience of having to wait to do my drugs to mail off a sample to have it tested. The main barrier for me is just that…I trust from a legal standpoint that this may all be confidential, what I don’t trust is that authority will respect that confidentiality. What I don’t trust is that this can’t be used to harm me. Especially in the new political climate. The idea of engaging with an app where I report substance use tied to my identity…knowing your rights is great, and knowing your legal protections is great. But, knowing them does not prevent authority from violating them…all knowing my rights does is gives me legal recourse after the fact to you know, try and pursue justice whether or not I receive it”.

Drug checking engagement involves balancing access and risk, where people weigh the convenience of getting quick, trusted information against possible social and legal harms. While participants showed strong interest in drug checking, especially when it is provided in familiar, low-barrier environments or directly to them, this willingness depends on reducing exposure to stigma, surveillance, and legal threats. Informal and private settings are often preferred not just for convenience but because they give more control over visibility and lessen perceived institutional dangers. Across different contexts, privacy and anonymity are crucial for engagement, particularly for those navigating intersecting stigmatized identities. Ultimately, even when services are available and acceptable, ongoing distrust in institutions and fear of punitive consequences limit participation. This suggests that expanding drug checking requires not only increasing access points but also fundamentally addressing the broader issues of criminalization and mistrust that influence how opportunities are perceived and acted upon.

### Motivation.

Motivational factors reflect complex, and sometimes competing, beliefs. Participants articulated strong beliefs about the consequences of drug checking, including perceived benefits, such as preventing overdose, avoiding fentanyl or xylazine exposure, and gaining greater knowledge of drug content.

The most obvious and acknowledged benefit was reducing overdoses from drug use. All participants had lost one or many people to what they felt could have been preventable overdoses. “Just this year I’ve had almost 5–6 of my friends die from Tina [Meth]…They were all together, so”. Another participant reflected on losing family members to overdose, and the role drug testing could have played to prevent those deaths.

“I’ve lost family members who OD’d, so I can understand. Drug tests would save lives. That is the major reason I can think of.” One participant reported losing as many as 20 people in the past two years. “You know how many friends I’ve lost over the last couple of years? About 25 down there [bus stop] to fentanyl and everything…at least 18 to 20 people that I know passed away down there within a 2-year period, if there is another good way to prevent overdoses then that is an important resource for the community”.

More than half of our participants expressed the desire to not only have the ability to identify contaminants, but also the ability to identify the full contents of their drugs. Fentanyl, Xylazine, and benzodiazepine test strips are single analytes. This acts as a barrier to motivation as it does not address people who use drugs’ desire to know the full contents of their drugs. FTIR and Spectrometry can provide a fuller picture but are currently inaccessible to those who use drugs in Fulton County.

“[FTS] helps because it’s like life or death…but then I think more people are curious as to like how much of this is real coke, and what is the average on that. Like if I get any bag in Atlanta right now no matter what side of Fulton County I get it on like what are we really talking…how many times is it getting stepped on and with what?”.

“Right, and you’re not sure of other drugs that might be in there because you’re testing for fentanyl specifically. So you don’t know if there’s something else in there that shouldn’t be in there, something to stretch out the quantity or to create a different high”.

Additionally, polysubstance use was common in our sample, and qualitative interviews contextualized polysubstance use as an intentional harm reduction strategy to manage intensity, duration, or adverse effects, complicating assumptions that drug checking decisions occur for single substances in isolation.

At the same time, distrust in law enforcement, concerns about data misuse, and fear of legal repercussions undermined the motivation to engage consistently, particularly with app-based or institutionalized testing models. One participant stated they would trust a drug checking app and “be inclined to use” it, however;

“the only barrier, the reason I would not consistently use it is because, I could even put up with the inconvenience of having to wait to do my drugs to mail off a sample to have it tested. The main barrier for me is just that…I trust from a legal standpoint that this may all be confidential, what I don’t trust is that authority will respect that confidentiality. What I don’t trust is that this can’t be used to harm me. Especially in the new political climate. The idea of engaging with an app where I report substance use tied to my identity…knowing your rights is great, and knowing your legal protections is great. But, knowing them does not prevent authority from violating them…all knowing my rights does is gives me legal recourse after the fact to you know, try and pursue justice whether or not I receive it”.

Optimism emerged in the belief that drug checking could save lives, especially among participants who had experienced a loss of friends or family to overdose. Emotional drivers, including grief, fear, and a sense of responsibility toward peers, were powerful motivators for testing and sharing results. Social influences also shaped motivation: trust in drug sources varied, with long-standing source relationships sometimes reducing the perceived need for testing, while distrust in the broader drug supply motivated interest in drug checking.

Trust in drug sources was generally high, with over half of the respondents reporting high trust in their dealer or source, and high trust in their dealer or source to provide the drug they were seeking (Table 3). As one participant notes, a trusted source is valuable and having to find a new source that can be trusted is common.

“I’m on my third or fourth reliable plug since I was 27 because they keep getting busted. I have someone who I really trust a lot right now, and they publish stuff about the testing that they do on each batch that they’re selling to us”.

Trust in drug sources emerged as a prominent quantitative finding and directly complemented the qualitative trust themes summarized in Table 4, derived from the option of “other reasons for not testing drugs”, providing an important context for understanding why many participants did not perceive a need for formal drug testing.

Conversely, concerns about technological trust and data privacy were common, with many respondents expressing fear of creating records of drug use or potential law enforcement access, underscoring the importance of privacy-preserving implementation approaches (Appendix A.1).

Incentives offered by researchers, public health departments, and harm reduction organizations to test drugs were identified as potential motivators, particularly for those experiencing economic precarity. Financial or material incentives for testing and result sharing were viewed as strategies that could increase participation, especially when testing required time, effort, or delayed use.

### Drug Checking Behaviors

When asked for reasons for not testing drugs, the most chosen responses were other reasons (59, 61.5%), I cannot find them locally (21, 21.9%), and too expensive (11, 11.5%). Nearly two-thirds of the respondents 96 (62.0%) reported that they had never tested their drugs. Among those who tested drugs (question offered to those who replied rarely, sometimes, often, and always to frequency of drug checking; n = 54), test strips were the most common method, particularly fentanyl test strips 41 (73.2%).

Analysis of open-ended responses and narratives revealed multiple reasons participants reported for not testing drugs prior to use. The most common explanations reflected trust-based decision-making, particularly reliance on a known dealer or source, with participants expressing confidence in their supplier’s honesty or consistency (e.g., “trust my dealer,” “I know who I get it from,” “dealer is honest”). Many respondents also described a low perceived need or risk, indicating that drug checking was not viewed as necessary or salient, often because it had never occurred to them or did not feel relevant to their use (“never thought about it,” “don’t feel the need,” “doesn’t seem necessary,” “I just don’t think about it”) (Table 4).

Habitual or routine use further contributed to non-testing, as participants described established consumption patterns in which testing was not incorporated (“it’s not something I do,” “same routine every time”). Others relied on informal or sensory assessment practices, such as visual inspection or taste, or cited familiarity with the substance, believing they already knew what the drug contained (“I tell by looking at it,” “by taste,” “by how it melts,” “I know what I’m getting,” “same stuff every time”) (Table 4).

## Discussion

### Trust as a Double-Edged Mechanism in Drug Checking

Trust has emerged as a central, yet paradoxical, mechanism for shaping engagement in drug checking. Consistent with prior research, interpersonal trust in drug sources, particularly long-standing relationships with dealers or friends, often functioned as a perceived harm reduction strategy, reducing participants’ perceived need to test drugs (49, 50). Participants in our study described trusting sources due to familiarity, consistent product quality, and mutual loyalty, echoing findings that trusted dealers may engage in informal consumer protection practices such as warning clients about strong batches or avoiding fentanyl-adulterated products (49). However, this reliance on trust simultaneously suppressed the motivation to engage in formal drug checking as several participants reported not testing drugs obtained from familiar sources. This finding aligns with emerging literature suggesting that trust can substitute for, rather than complement, technological harm-reduction strategies (49, 51). While trust in sources was perceived as protective, participants also acknowledged its limits, noting that risk is “never 100% eliminated.” This tension underscores trust as a double-edged mechanism that can reduce perceived risk while potentially obscuring exposure to an increasingly unpredictable drug supply.

In contrast, institutional trust, particularly in harm-reduction organizations, facilitates drug-checking participation. Participants expressed strong confidence in trusted harm reduction agencies as appropriate sites for advanced technologies, such as FTIR, aligning with prior work demonstrating that trust, transparency, and anonymity are critical to the acceptability of drug checking services (52, 53). This distinction between interpersonal and institutional trust highlights the need for implementation strategies that do not directly challenge trusted social relationships but instead integrate drug checking into existing community networks, reinforcing rather than undermining social cohesion.

### Polysubstance Use and the Limits of Single-Substance Drug Checking

Intentional polysubstance use was pervasive in both our qualitative and quantitative findings and presented a critical challenge to current drug-checking models. Participants frequently described combining stimulants and depressants to modulate effects, manage withdrawal, enhance pleasure, or mitigate perceived risks, patterns well-documented in the literature (54–56). These practices are often framed as purposeful and adaptive, rather than accidental, reflecting sophisticated experiential knowledge of drug effects.

Our findings reinforce the concern that single-analyte testing approaches, such as fentanyl-only test strips, may inadequately capture the lived realities of drug use. Participants expressed frustration with binary results that failed to provide information on concentration, purity, or the presence of additional adulterants, concerns echoed by science parks and drug-checking experts, who emphasize the limitations of rapid immunoassay testing (11, 57). This gap may reduce the perceived utility of testing among people who intentionally combine substances, particularly when the test results do not align with experiential effects.

Advanced drug-checking technologies, such as FTIR, were viewed by participants as better aligned with their informational needs, offering the potential for multi-analyte detection and greater clarity regarding drug composition. This aligns with evidence from recreational and community-based settings, demonstrating that more comprehensive testing methods can influence decision-making, particularly when results are ambiguous or unexpected (58). Importantly, participants in our study emphasized that understanding “what else is in the mix” was as important as detecting fentanyl alone, underscoring the need for drug-checking interventions that reflect real-world polysubstance use patterns rather than idealized single-drug scenarios.

### Place, Context, and the Southern U.S. Implementation Landscape

Place has emerged as a critical determinant of drug use and drug checking behavior. Consistent with prior studies, home use and public or semi-public spaces (e.g., streets, cars, bathrooms) were common settings (59–61). However, our findings extend the literature by highlighting substantial drug use in social and nightlife settings, such as parties, bars, clubs, and concerts, contexts largely absent from prior research focused on injection or street-based use. These environments present unique opportunities for community-based drug checking but also raise concerns related to visibility, stigma, and enforcement.

Implementation barriers were particularly salient in the Southern U.S. context. Despite recent legislative changes in Georgia enabling drug checking, participants expressed a persistent fear of law enforcement surveillance and arrest, reflecting a legacy of punitive drug policy and structural mistrust in the region, similar to mistrust seen in other studies with people who use drugs (62). These concerns mirror findings from other countries, where criminalization produces structural stigma that discourages engagement with harm-reduction services, even when legal protections exist (52).

Participants’ skepticism that law enforcement would respect drug-checking protections underscores the limitations of policy changes alone. As prior work suggests, implementation success depends not only on legality but also on perceived legitimacy and safety (63, 64). Community-led, peer-driven models embedded within trusted harm reduction organizations may be particularly critical in the Southern U.S., where historical marginalization, racialized policing, and institutional distrust shape health behaviors. Our findings suggest that drug-checking services must be visibly independent of law enforcement, prioritize anonymity, and be delivered by individuals with lived experience to overcome these deeply rooted barriers.

### Implications for Harm Reduction and Drug Checking Interventions

Taken together, these findings support the expanded conceptualization of drug checking as more than a technical intervention. Consistent with prior qualitative work, participants viewed drug checking as a tool for empowerment, community care, and informal market regulation, rather than solely overdose prevention (65). Trust, place, and polysubstance use interact to shape whether drug checking is perceived as relevant, feasible, or worthy of effort.

Interventions that fail to account for these dynamics, by emphasizing individual responsibility, single-drug risk, or institutional surveillance, have risk-limited uptake. Conversely, drug-checking models that acknowledge lived drug use practices, leverage trusted community relationships, and offer meaningful, actionable information may better align with the realities of contemporary drug use and contribute to reductions in morbidity and mortality associated with an increasingly adulterated drug supply.

### Strengths and Limitations

This study has several notable strengths. First, its community-driven, community-based participatory approach intentionally engaged people who use drugs throughout every phase of the research process, from study design through implementation and interpretation. The use of trusted community members as community liaisons for participant recruitment facilitated access to individuals who may otherwise have been underrepresented in research, particularly Black and African American people who use drugs. This recruitment strategy strengthened trust, enhanced the relevance of the findings, and improved the representativeness of participant perspectives.

Although not reported in this manuscript, the research team also made a substantial investment in capacity building among people with lived and living experience who contributed to the study, strengthening community research infrastructure and fostering equitable research partnerships.

To our knowledge, this is among the first studies to apply the COM-B model to examine drug checking behaviors among people who use drugs in the southeastern United States. Designing the study using this well-established behavioral science framework provided a rigorous foundation for understanding the capability, opportunity, and motivational factors influencing engagement with drug-checking services. Rather than simply identifying barriers and facilitators, the COM-B framework enabled the identification of modifiable behavioral determinants that can directly inform the design of future interventions aimed at increasing drug checking uptake.

Additionally, the inclusion of participants with diverse lived experiences of substance use, overdose, and harm reduction enhances the transferability of the findings to community-based harm reduction settings serving similarly marginalized populations. Finally, the use of rigorous qualitative methods, including systematic coding and collaborative analysis, strengthens the trustworthiness and credibility of the findings while providing a clear pathway for future theory-informed intervention development and implementation.

Several limitations of this study should be considered when interpreting these findings. First, the study relied on self-reported data, which may be subject to recall or social desirability bias, particularly given the stigmatized and criminalized nature of drug use. Although efforts were made to foster trust and confidentiality, participants may have underreported certain behaviors (e.g., drug use in highly visible settings) or overreported perceived harm reduction practices.

Second, while the mixed-methods design strengthens interpretation, the quantitative survey did not capture all dimensions of polysubstance use (e.g., simultaneous versus sequential use, dosage, or timing), nor did it fully integrate alcohol, tobacco, or cannabis into the polysubstance analytic framework. Consequently, some nuances of the co-use patterns described in qualitative interviews could not be fully quantified.

Third, the study was conducted in an urban Southern U.S. context and may not be generalizable to rural settings, other regions, or jurisdictions with different drug policies and harm reduction infrastructure. However, this context-specific focus is also a strength, as much of the existing drug-checking literature is concentrated in the Northeast, West Coast, or international settings. Finally, while participants discussed advanced drug checking technologies, such as FTIR and app-based systems, most did not have direct experience using these tools, and perceptions were therefore hypothetical. Future research should evaluate how stated intentions translate into real-world engagement when such services are implemented.

### Findings Within COM-B Framework

From a COM-B perspective, drug-checking behavior is shaped by the interaction of capability, opportunity, and motivation. Capability is influenced by uneven knowledge of evidence-based drug checking methods and reliance on experiential or sensory testing practices. Skills and behavioral regulation were constrained by the urgency to use drugs, withdrawal, and cognitive effects of substance use. Opportunity is shaped by environmental access to testing tools, availability of trusted locations, and social conditions such as stigma, privacy, and fear of law enforcement. Motivation reflected competing beliefs: while many participants believed that drug checking could save lives, trust in familiar drug sources and skepticism about institutional safety reduced the perceived necessity.

### Capability: Self-Efficacy, Survival Orientation, and Drug Checking

Findings suggest that psychological capability to engage in drug checking is strongly shaped by self-efficacy and by the role drugs play in meeting immediate survival needs. Participants who described low self-efficacy, often in the context of homelessness, trauma exposure, health deterioration, or physical and psychological dependence, were less likely to report engagement in drug checking. For these individuals, drug use was frequently framed as necessary for coping or survival, limiting the perceived feasibility of delaying use for testing. In contrast, participants who described greater control over their drug use and higher self-efficacy, often characterized by recreational or situational use, were more likely to report prior drug checking and openness to future engagement, including use of a drug-checking application. Prior experience with drug checking emerged as both an indicator of capability and a facilitator of continued engagement, reinforcing the role of experiential learning in building confidence. Importantly, capability appeared to exist along a spectrum. Engagement in drug checking varied according to where drug use fell within participants’ hierarchy of needs, suggesting that interventions must account for differential capacity rather than assuming uniform readiness across people who use drugs.

### Opportunity: Trust, Place, and the Limits of Access Alone

Opportunity emerged as a central determinant of drug checking engagement, particularly through the intersecting roles of trust and place. Although participants frequently identified public spaces, such as libraries, public health departments, and pharmacies, as theoretically ideal locations for drug checking due to accessibility and convenience, quantitative findings did not support these preferences. Instead, fear of law enforcement surveillance, stigma, and visibility in public spaces substantially constrained willingness to engage. These findings suggest that technically accessible locations may function as perceived barriers when trust is low. Implementing drug checking services in public or institutional settings without a parallel educational and destigmatization campaign may inadvertently reinforce fear and avoidance, even when such services are legally supported. Access to drug checking tools alone was insufficient to facilitate engagement. Despite reported availability of fentanyl and xylazine test strips, particularly among participants connected to syringe services programs, access did not reliably translate into use. Across both survey and interview data, participants emphasized the need for education on how, when, and why to test drugs, highlighting opportunity as a function of knowledge, safety, and trust rather than mere physical availability.

### Evolving Needs: Technology and Education

Participants identified a clear need for the evolution of drug checking technologies beyond single-analyte test strips. While fentanyl and xylazine test strips were viewed as valuable screening tools, participants expressed frustration with their limited specificity and inability to provide information on concentration or multiple substances. Several participants noted that test strips were designed for urine samples and may produce misleading results when used with drug residues, reducing confidence in their accuracy.

Advanced drug checking technologies, such as Fourier-transform infrared spectroscopy (FTIR), were perceived as better aligned with participants’ informational needs, particularly in the context of intentional polysubstance use. These findings reinforce the importance of adapting drug checking infrastructure to evolving drug markets and user priorities. Equally critical was the need for comprehensive drug education. Despite recent policy changes and the expansion of harm reduction services in Fulton County, many participants lacked awareness of available resources, legal protections, and evidence-based harm reduction strategies. This educational gap extended beyond people who use drugs to include stakeholders and service providers, underscoring the importance of parallel training and community-wide education to support effective implementation.

COM-B highlights that increasing drug uptake requires more than education or tool distribution. Effective interventions must simultaneously enhance individual capability, expand safe and trusted opportunities, and address motivations while being implemented through community-centered systems that provide legal protections and are responsive to the needs of the PWUD.

### Implications for Intervention Design

Mapping the COM-B and TDF domains to the Behavior Change Wheel intervention functions provides a clear roadmap for intervention development. Effective strategies should combine education and training to build capability, environmental restructuring to enhance opportunity, and persuasive peer-led messaging to strengthen motivation. Drug checking should be framed as a complementary layer of harm reduction rather than a replacement for existing strategies.

## Conclusion

This study demonstrates that while community drug checking is urgently needed in Fulton County, its successful implementation is constrained by stigma, trust in source, and limited drug education. Legal reforms and expanded access to testing tools alone are insufficient to ensure uptake of testing strips. Instead, effective drug checking interventions must be embedded within trusted community settings, supported by education and destigmatization efforts, and responsive to the realities of polysubstance use. In a resource-constrained and historically punitive policy environment, investments in education, community liaison infrastructure, and adaptive drug checking technologies are essential for meaningful public health impact.

## Supplementary Material

This is a list of supplementary files associated with this preprint. Click to download.

- Supplement1.docx
- AppendixA.docx

## Figures and Tables

**Figure 1:**
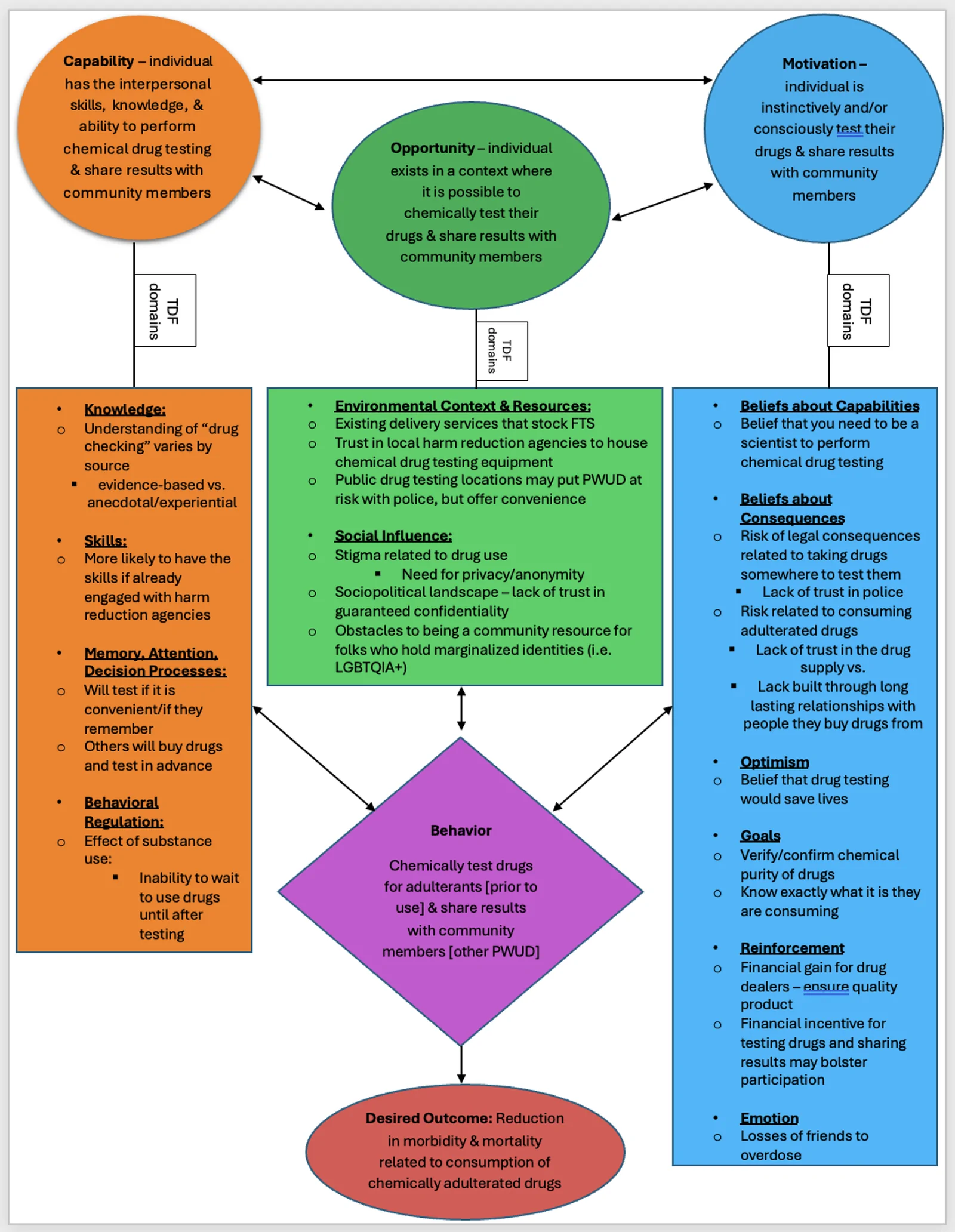
COM-B and TDF framework integration, adapted from Jatau et al. (2019).

**Table 1:** Participant Characteristics (N = 164)

Characteristic	Level	Overall
Age, mean (SD)	—	41.72 (13.81)
Sex, n (%)	Female	57 (35.4)
	Male	104 (64.6)
Race, n (%)	Asian	2 (1.2)
	Other/Unknown	5 (3.0)
	White	26 (15.9)
	Black or African American	131 (79.9)
Education, n (%)	Graduate Degree	4 (2.5)
	Associate’s Degree	9 (5.5)
	Bachelor’s Degree	20 (12.3)
	Some High School	29 (17.8)
	Some College	39 (23.9)
	High School/GED	57 (35.0)
Employment, n (%)	Employed part-time	22 (13.5)
	Self-employed	30 (18.4)
	Employed full-time	36 (22.1)
	Unemployed	57 (35.0)
Syringe Service Program Use, n (%)	Yes	37 (23.0)
	No	124 (77.0)

**Table 2:** Drug Use settings

Category	n (%)
**Drug Use Settings in the Past 12 Months; Select all that apply (N = 158)**	
At home	114 (72.2)
Parties	73 (46.2)
Bars/clubs	71 (44.9)
On the street	66 (41.8)
In cars	61 (38.6)
Concerts	47 (29.7)
Public bathrooms	44 (27.8)
Restaurants	31 (19.6)
At school	9 (5.7)
Libraries	7 (4.4)

**Table 3:** Trust in Drug Source

Trust Measure	Level	n (%)
Trust in drug dealer/source (1–10 scale)	High trust (8–10)	91 (56.9)
	Moderate trust (4–7)	55 (34.4)
	Low trust (1–3)	14 (8.8)
Trust dealer/source will sell intended drug (1–10 scale)	High trust (8–10)	95 (60.5)
	Moderate trust (4–7)	44 (28.0)
	Low trust (1–3)	18 (11.5)

**Table 4:** Coding of open-ended responses describing “other reasons for not testing drugs.”

Code	Category Name	Definition	Example Response Types
1	Trust in source / dealer-based trust	Reliance on a dealer or supplier as a reason for not testing drugs.	“Trust my dealer”; “I know who I get it from”; “Dealer is honest”
2	Low perceived need / low perceived risk	Statements indicating testing is unnecessary or not salient.	“Never thought about it”; “Don’t feel the need”; “Doesn’t seem necessary”
3	Habitual or routine use	Testing not incorporated into routine or habitual drug use.	“I just don’t think about it”; “It’s not something I do”; “Same routine every time”
4	Informal or sensory assessment practices	Use of sensory or experiential cues instead of formal testing.	“I can tell by looking at it”; “By taste”; “By how it melts”
5	Familiarity with substance or expected contents	Belief that drug contents are predictable or already known.	“I know what I’m getting”; “Same stuff every time”
6	Lack of knowledge about testing	Uncertainty about how to test or how testing works.	“Don’t know how to test”; “Not sure how it works”
7	Access or availability barriers	Difficulty obtaining or locating drug-checking supplies.	“Hard to find”; “Not available where I am”
8	Convenience / effort barriers	Testing perceived as inconvenient or requiring too much effort.	“Too much work”; “Extra step”; “Don’t want to deal with it”
9	Alternative risk-reduction strategies	Use of other strategies instead of testing to manage risk.	“I smoke first”; “I start small”; “I go slow”
10	Other / idiosyncratic	Unique or unclear responses not fitting other categories.	Highly specific or ambiguous responses

## Data Availability

The datasets generated and analyzed during the current study are not publicly available due to ethical restrictions related to the sensitive nature of illicit drug use data and the risk of participant re-identification. De-identified quantitative and qualitative data may be made available upon reasonable request to the corresponding author and with approval from the Emory University Institutional Review Board.

## Abbreviations

US: United States
COM: B-Capability, Opportunity, Motivation-Behavior
PWUD: People Who use Drugs
DCS: Drug checking services
FTS: Fentanyl Test Strips
XTS: Xylazine Test Strips
FTIR: Fourier-transform infrared spectroscopy
SSP: Syringe Services Program
TDF: The Theoretical Domains Framework
BCW: Behavior Change Wheel
